# Medicine, Big Business, and Public Health: Wake Up and Smell the Starbucks

**Published:** 2009-03-15

**Authors:** Eileen Salinsky

**Affiliations:** AARP, Office of the CEO

## Abstract

The provision of ambulatory care by major retailers is small but growing, providing speedy attention to consumers with minimal wait times and no appointments necessary. Users of these clinics are satisfied with the care they receive. Primary care physicians have opposed retail clinics, concerned that conditions will be misdiagnosed, opportunities to address comorbidities and risk behaviors will be missed, necessary follow-up care will be delayed or absent, and the profit motive will lead to cutting corners. Public health is now being challenged to capitalize on the advantageous possibilities these clinics can offer, such as serving uninsured patients, while remaining vigilant regarding potential hazards, such as financial pressures that could negatively affect health care quality, continuity, and accessibility.

## Background

Public health has long maintained a sibling rivalry of sorts with its flashier, more popular sister — medical care. Population-based, prevention-oriented interventions are often overshadowed by medical treatments geared toward the curative needs of individual patients. The widely cited statistic that public health accounts for less than 3% of health-related spending in the United States (1) is frequently used to illustrate the paucity of resources and attention given to public health compared with the riches and prestige bestowed on medical science.

**Table:** 

**Box. Synergistic Strategies That Characterize Common Forms of Medical–Public Health Partnership^a^**
Improve access to care for the uninsured and underinsured. Use clinical practice to identify and address community health problems (such as disease surveillance and vaccination activities). Coordinate medical care for individuals with support services commonly provided by public health agencies (such as nurse home visitation programs). Improve the quality and cost-effectiveness of medical care by applying a population perspective (such as using population-based information to enhance clinical decision making). Strengthen disease prevention and health promotion by mobilizing community campaigns (such as mounting health education efforts). Shape health system development (such as policy advocacy activities).

a Source: Lasker et al (2).

Despite some latent jealousies and important differences in priorities and perspectives, public health workers have historically collaborated with physicians and other health care providers to improve the health of individuals and communities. The nature and strength of this partnership has evolved over the years but has been characterized by a mutual recognition of the utility, value, and interdependency of the medical and public health models (Box) (2). Some worry that time-tested collaborative endeavors are growing strained as corporate interests increasingly drive practices and priorities in the health care industry. This corporatization is perhaps most vividly demonstrated by the small but growing presence of major retailers in the provision of ambulatory care. These "convenience" or "retail" clinics are in retail commercial outlets (such as discount superstores, grocery stores, and pharmacies), typically offer a limited scope of services, are often staffed by a nurse practitioner, and provide speedy attention to consumers with minimal wait times and no appointments necessary. Clinics rent retail space from the "host" retailer and are usually owned by independent clinic operator organizations (such as MinuteClinic) or by conventional health care systems. In 2007, approximately 500 retail clinics operated in 36 states, and their numbers are expected to swell in the near future (3). Conservative estimates suggest that 2,500 retail clinics will be operational in the next 4 years, while more ambitious forecasts predict approximately 6,000 retail clinic sites in 2012 (4).

## Characteristics of Retail Clinic Users

Clinic sponsors and other proponents argue that retail clinics provide needed (and carefully targeted) services in a responsive and cost-efficient manner to people who would otherwise have limited access to health care (5). Evidence indicates that users are highly satisfied with the retail clinic experience. A 2008 Harris poll revealed that 90% of clinic users were happy with the quality of care provided (6). Although only a small proportion of respondents visited clinics (7%), consumer acceptance of the clinic model is growing, with just 65% of respondents expressing some level of wariness regarding provider qualifications, down from 71% in 2005 (6).

Retail clinic users also appear to improve access for the underserved. Mehrotra et al (4) found that retail clinic patients are predominantly young adults (43% of clinic patients), are unlikely to have a regular primary care provider (61.3% of clinic patients), and are more likely to pay out of pocket (32.9% of clinic patients) than patients who visit a primary care provider (9.9% of primary care provider patients) or the emergency department (24.6% of emergency department patients) (4). Out-of-pocket payment is an imperfect proxy for insurance status, as some clinics do not accept insurance payments and some insurers do not cover retail clinic visits. However, the Harris poll results confirm that a substantial proportion of retail clinic users (16%) are uninsured (6).

## Concerns About Retail Clinics

Primary care physicians have been quite vocal in their opposition to retail clinics (4). Concerns center on fears that conditions will be misdiagnosed, opportunities to address comorbidities and risk behaviors will be missed, necessary follow-up care will be delayed or absent, and the profit motive will lead to cutting corners and providing insufficient service to the patient. Some worry that retail clinics will "skim" the straightforward and sometimes lucrative patients from more traditional primary care providers and undercut the financial viability of full-service care sites. This concern is particularly great for safety net clinics that operate with very thin financial margins. Retail clinics serve a large number of uninsured patients (6), but they are not likely to deliver free and reduced-price services in the manner of community health centers and other safety net providers. This suggests that retail clinics are not a dependable care alternative for the poorest and most vulnerable patients. Another reason that retail clinics are not dependable access enhancers is that retail clinics will exit markets quickly if expected financial returns are not forthcoming, potentially leaving regular customers without a source of care.

Concerns that financial imperatives will negatively affect health care quality, continuity, and accessibility are by no means unprecedented, but they take on renewed poignancy in light of the disruptive innovation potentially posed by retail clinics. Similar issues were raised as managed care became more prevalent and imposed escalating financial pressure on health care providers (7). The current dynamic echoes past efforts to promote cost-efficiency, but recent innovations portend a more radical restructuring of the provider-patient relationship and a shift in the character of public health's medical partner. However, the disruptive potential of McMedicine clinics remains largely speculative (8).

The real effect of retail clinics on patient care, and on population-based services that intersect with medicine, depends on how widely this care model is used and the ways in which these clinics relate to conventional primary care and public health. In addition to worrying about jeopardizing the quality and continuity of patient care and the financial health of existing safety net providers, public health professionals also worry about less publicized issues, such as disease surveillance compliance, disease screening opportunities, health education capacities, and appropriate use of antibiotics. Will retail clinics participate in vaccination registries? Assess conjugate pneumococcal vaccination status for children with otitis media? Comply with influenza vaccine recommendations in the event of a shortage? Provide appropriate antibiotic treatment? Convey tobacco use cessation guidance to adult patients with pharyngitis? Recommend diabetes screening to the overweight patient with a urinary tract infection? These specific inquiries raise the larger question: Are partnerships with public health compatible with the retail clinic business model?

Tensions between profit motives and humanitarian goals in health care are not new. Private outpatient medical practices have always operated as small businesses, but traditionally the professional ethics of independent health care practitioners have been perceived to mediate the potentially pernicious influence of commerce (7). Prevailing reimbursement incentives make chronic disease good for business if your business is medicine. Health care providers' willingness to contribute to disease prevention activities is rooted in their professional ethos, not their monetary objectives.

As the independence and decision-making authority of individual professionals are diminished within large corporate bureaucracies, critics worry that both the quality of patient care and commitment to the public good will suffer. Some limited evidence exists to support these concerns. For example, physicians with an ownership stake in their practice are more likely to provide charity care than those employed by a private practice (9).

## Retail Clinic Characteristics and Public Health

Public health has an important role to play in monitoring the consequences of changing health care delivery models and organizational structures, but appropriate watchfulness should not prevent public health officials from exploring and initiating collaborative opportunities with these new commercial partners. As the nature of medical practice evolves, public health must continue to seek ways to harness the reach and creativity of new corporate stakeholders. These opportunities are likely to take on a variety of forms — some will prove viable, while others will fail to be realized.

In many respects, the goals and structure of retail clinics appear to align quite well with public health objectives. Retail clinics have the potential to make a variety of value-added contributions to the traditional medical–public health partnership. Several characteristics of these care sites could be marshaled to improve population health outcomes.

### Consumer orientation

The retail clinic business model is firmly grounded in a sophisticated understanding of what consumers want and how they make decisions. The marketing advantages of on-site clinical services may be more appealing to retailers than the discrete profits these clinics generate. These services draw customers to the retail location, trigger demand for related products such as pharmaceuticals and over-the-counter drugs, and help establish a "wellness" brand for the retailer. Retailers invest heavily in multiple forms of marketing, including mass media advertising, direct mailings, in-store promotions, coupons, and niche marketing techniques. Public health can explore ways to leverage these marketing goals and competencies to design and implement collaborative social marketing campaigns related to clinical preventive services, risk behaviors, and health promotion messages.

These types of public–private partnerships have a proven track record in health promotion. For example, the Back to Sleep campaign to reduce sudden infant death syndrome received a substantial boost from Proctor and Gamble, which contributed marketing expertise to the campaign and aided in message dissemination (10). Retail clinics and their retail sponsors may be highly motivated to participate in these types of partnerships with public health. The credibility and positive publicity of health promotion activities aid in cultivating a wellness brand and inspire consumer confidence.

### Information technology resources

To maintain an acute awareness of consumer preferences, retail businesses invest substantial resources in information technology and data-gathering activities. These efforts are oriented toward who buys what where, when, and why. But these consumer monitoring techniques (such as customer surveys, focus groups, purchasing profiles, and frequent shopper programs) also have the potential to provide valuable information for public health research and practice.

Public health has begun piloting methods to use consumer product data for syndromic surveillance. Additional forms of data sharing and collaborative monitoring hold tremendous promise. For example, most retail clinics use electronic medical records. These electronic data can be shared with conventional primary care providers and incorporated into existing registries and emerging surveillance methods while protecting patient privacy. Another example is self-guided patient education through computer kiosks, which has been tested in physician and social service offices and could be adapted for the retail clinic setting.

### Workforce competencies

Retail clinics are typically staffed by nurse practitioners, and clinic operators have noted that the quality and communication skills of the clinical staff are central to achieving consumer satisfaction. The premium placed on employing highly competent clinicians with superior interpersonal skills suggests that these retail clinic employees could be valuable allies in disseminating disease prevention and health promotion messages.

### Market penetration

Retail distributors have tremendous reach given their strategic locations proximate to population growth centers. They are highly efficient in inventory tracking and management. Mass merchandisers analyze customer flow and movement throughout their stores and are skilled in directing foot traffic toward promotional displays. These skills could be used in planning for emergency mass prophylaxis and for carrying out routine, universal treatment services such as annual influenza vaccination campaigns.

### Influence with policy makers

Corporate interests represent an influential constituency for public policy makers. Inculcating public health priorities into the public policy agenda of corporate stakeholders substantially improves the likelihood that these objectives will be achieved.

## Conclusion

Some healthy skepticism is warranted in the pursuit of these opportunities. Public health advocates should remain mindful that corporate partners are primarily driven by profit motives. But strong communication and interactive exchange between public health and commercialized medicine can reveal "win-win" opportunities. Corporate branding and public relations goals may align well with health improvement objectives. Some public health professionals may find it challenging to make peace with their own reluctance to acknowledge the legitimacy of commercial interests. Similarly, retail partners may need assistance in overcoming their own perceptions about the bureaucratic burdens of working with government agencies. Competitive tensions among corporate partners must also be acknowledged. Public health will need to be receptive to all interested, appropriate partners and avoid exclusive partnering arrangements.

The history of public health is defined by the field's ability to adapt and respond to the evolving threats to human health that have emanated from a changing environment. The health care industry's increasing emphasis on financial returns (which transcends the emergence of retail clinics) and the proliferation of new care delivery models pose new threats and opportunities for public health. The field is now being challenged to capitalize on advantageous possibilities while remaining vigilant regarding potential hazards. Such dexterity will require both open minds and open eyes; ready for that macchiato?
